# Immune-Mediated Necrotizing Myopathy (IMNM): A Story of Antibodies

**DOI:** 10.3390/antib13010012

**Published:** 2024-02-07

**Authors:** Sarah Julien, Inès Challier, Marine Malleter, Fabienne Jouen, Laurent Drouot, Olivier Boyer

**Affiliations:** 1 INSERM U1234, PAn’THER FOCIS Center of Excellence, Université de Rouen, F-76000 Rouen, France; sarah.julien@univ-rouen.fr (S.J.); marine.malleter@univ-rouen.fr (M.M.); fabienne.jouen@chu-rouen.fr (F.J.); laurent.drouot@univ-rouen.fr (L.D.); 2 Department of Pharmacy, CHU Rouen, F-76000 Rouen, France; ines.challier@univ-rouen.fr; 3 Department of Immunology and Biotherapy, CHU Rouen, F-76000 Rouen, France

**Keywords:** immune-mediated necrotizing myopathy, myositis, anti-SRP, anti-HMGCR, autoantibodies

## Abstract

Immune-mediated necrotizing myopathy (IMNM) is a rare and severe disease that corresponds to a specific entity of idiopathic inflammatory myopathy. Patients with IMNM suffer from proximal muscle weakness, and present high levels of creatine kinase and necrotic myofibers. Anti-Signal Recognition Particle (SRP) and anti-3-hydroxy-3-methylglutaryl-coenzyme A reductase autoantibodies (HMGCR) have recently been identified in two thirds of patients with IMNM and are used as a hallmark of the disease. In this review, we provide a detailed description of these antibodies and the tests used to detect them in the serum of patients. Based on in vitro studies and mouse models of IMNM, we discuss the role of autoantibodies in the pathogenesis of the disease. Finally, in the light of the latest knowledge, we conclude with a review of recent therapeutic approaches in IMNM.

## 1. Introduction

Idiopathic inflammatory myopathies (IIM), commonly known as myositis, are a group of rare muscle autoimmune diseases characterized by a heterogeneous clinical presentation. The most common clinical manifestations include acute or subacute muscle weakness, limited muscle endurance, and myalgia associated with chronic muscle inflammation.

The initial classification criteria were established and published by Bohan and Peter in 1975. This classification focuses on two different types of disease: polymyositis (PM) and dermatomyositis (DM). It is based on clinical and histological criteria and takes into account serum levels of muscle enzymes including creatine kinase (CK) [1,2]. In 2003, the 119th international workshop of the European Neuromuscular Center (ENMC) proposed new classification criteria, highlighting several subtypes of myositis. There are five different types: inclusion body myositis (IBM), PM, DM, non-specific myositis with perimysial and perivascular infiltrates distinct from PM and DM, and finally immune-mediated necrotizing myopathy (IMNM) without infiltrates [3]. In IIM, 60% of cases are associated with the presence of autoantibodies (aAbs). Multiple antibodies are found in each subtype of myositis, i.e., Myositis Specific Autoantibodies (MSA) and they are generally mutually exclusive allowing the distinction between the different types of myositis and a new classification [4]. Then, in 2017, the updated classification criteria of the European League Against Rheumatism and the American College of Rheumatology (EULAR/ACR) were published. Ninety-three variables including disease course, pattern of weakness, dermatological manifestations, systemic manifestations, response to treatment, muscle biopsy abnormalities, electro-myogram (EMG), magnetic resonance imaging (MRI) and presence of anti-JO1 MSA were identified [5,6]. Finally, in 2018, a classification system for IIM, including MSA, was proposed [7]. A retrospective study, based on data from 260 French patients from the French Myositis Network, collected from 2003 to 2016, led to the establishment of a classification based on four distinct types of myositis: IBM, IMNM, DM and anti-synthetase syndrome (ASA). IBM affects white male patients over 60 years of age and is characterized by weakness in the finger flexors and quadriceps, with abnormalities on muscle biopsy such as vacuoles and mitochondrial fiber alterations. IMNM mainly affects women and is characterized by elevated CK levels, muscle necrosis without significant inflammation, and the presence of MSA anti-Signal Recognition Particle (SRP) or anti-3-hydroxy-3-methylglutaryl-coenzyme A reductase (HMGCR). DM is characterized by skin rash and antibodies against Mi2, MDA5 (melanoma differentiation-associated protein 5), or TIF1γ (transcription intermediary factor-1γ). Finally, ASA is characterized by the presence of anti-Jo1, anti-PL7 or other anti-tRNA synthetase antibodies [7]. The integration of MSA into this classification represented a significant advance in the diagnosis of these diseases.

Based on the presence of aAbs, IMNM can be divided in three groups: anti-SRP positive, anti-HMGCR positive and seronegative IMNM. Each group shares common features and could be characterized by proximal acute or subacute muscle weakness with a symmetric distribution, sometimes accompanied by myalgia [8]. A study published in 2017 reported more severe muscular manifestations in patients with anti-SRP positive compared to anti-HMGCR positive or seronegative IMNM [9]. These patients are also more prone to developing cardiac involvement, interstitial lung disease (ILD), and dysphagia [10]. On the other hand, patients with anti-HMGCR positive IMNM mainly present with muscular weakness without extra-muscular involvement. Seronegative IMNM is still relatively understudied today. However, it appears that patients with seronegative IMNM exhibit a higher risk of cancer compared to those classified as seropositive [11].

## 2. Anti-SRP and Anti-HMGCR Autoantibody as a Hallmark of Disease

Anti-SRP aAbs were first discovered in 1986 by Reeves et al. in patients diagnosed with PM [12]. The Signal Recognition Particle (SRP) is a complex composed of six proteins, SRP9, SRP14, SRP19, SRP54, SRP68, SRP72, and 7SL RNA respectively. This complex, along with its receptor (SR), facilitates the translocation of neo synthetized proteins from the cytosol to the endoplasmic reticulum (ER). The process occurs in three steps. First, while the protein is translated by the ribosome, SRP54 recognizes the hydrophobic signal sequence of the protein. With the involvement of 7SL RNA, it stops in a transitory manner its elongation. Then the SRP complex binds to its receptor on the ER membrane. Finally, GTP hydrolysis, which occurs following interaction with the SR, releases the SRP from the ribosome-signal peptide complex, allowing it to bind to another signal peptide. Protein translation then resumes at the translocation channel, called sec61, guiding the protein into the lumen of the ER [13]. Anti-SRP54 aAbs are found in 15% of diagnosed patients [8]. We demonstrated in 21 sera from patients with anti-SRP positive IMNM that 81% of the patients had isotype 1 IgG antibodies, and 71% of these IgG1 antibodies were present alone while the others were associated with one or two other isotypes. IgG4 was found in 29% of the patients, while IgG3 was only found in one sample, and IgG2 was not detected anywhere [14]. Four different antibodies associated with IMNM were identified and target SRP19, SRP54, SRP72 proteins and 7SL RNA [15,16,17,18]. Anti-SRP54 is the most dominant antibody found in patients with anti-SRP IMNM. It has been shown that anti-SRP54 antibodies can bind to the amino-terminal SRP54 N-domain and the central SRP54 G-domain. However, they cannot bind to the carboxy-terminal M-domain which is responsible for binding ER signal sequences [16]. To our knowledge, the epitope mapping of other anti-SRP aAbs has not been performed.

Anti-HMGCR aAbs were discovered in 2010 by Christopher-Stine et al. [19]. HMGCR was initially considered as a target of aAbs in patients with IMNM due to evidence that several individuals developed IMNM following statin exposure [20,21]. Statins are used as medications to lower cholesterol levels by targeting HMGCR, a protein involved in the synthesis of cholesterol. HMGCR is a 97 kDa glycoprotein localized in the ER. This enzyme contains a hydrophobic N-terminal domain that crosses the ER membrane and a soluble domain into the cytosol. The C-terminal domain is the effector domain of the enzyme and catalyzes the transformation of HMG-CoA to mevalonate, the precursor of cholesterol [22]. The anti-HMGCR antibody has been identified in more than 60% of cohorts in some studies [10]. It has been demonstrated that anti-HMGCR aAbs recognize a portion of the protein spanning a part of the C-terminal domain and intracellular portion (amino acids 340 to 888) [23].

Seronegative IMNM, described as IMNM without anti-SRP or anti-HMGCR aAbs, represents between 10 and 12% of patients [10]. Allenbach et al. described this subclass “by default” with poorly studied characteristics [10]. The diagnosis of seronegative patients remains a difficult task. Possibly, sera from these patients with seronegative IMNM contain an undiscovered autoantibody.

## 3. Autoantibody Assays at the Heart of Diagnosis

To diagnose IIM, aAbs are systematically tested. Several methods are available to detect aAbs: immunoprecipitation (IP), line blot, dot blot, enzyme-linked immunosorbent assay (ELISA), Addressable Laser Bead ImmunoAssay (ALBIA), or chemiluminescence assay (CLIA) and they could be completed by Indirect Immunofluorescence (IFI) on human epithelial type 2 (Hep2) cells.

The reference method for detecting IIM autoantibodies is IP. This method has allowed the discovery of many specific aAbs in autoimmune diseases as anti-SRP and anti-HMGCR aAbs in IMNM [12,19]. Moreover, IP is sensitive and specific. However, the use of IP in routine is not relevant because it is rarely available in medical biology laboratories [24]. Several detection methods are now available in routine practice. aAbs can be detected by mono- or multispecific (detection of multiple antibodies at the same time) immunoassays [25]. These methods can be qualitative and/or quantitative. Commercial multispecific immunoassays correspond to immunoblot (line or dot blot) and are qualitative. Their main advantage is being able to detect all specific myositis antibodies in a single test run. In contrast, monospecific immunoassays are quantitative. They also have excellent analytical and diagnostic performance. However, their disadvantage is that they only allow the detection of one antibody at a time. ELISA and ALBIA tests using SRP54 as antigen are used to detect anti-SRP aAbs, and ELISA, CLIA and ALBIA for detecting anti-HMGCR aAbs. Only ELISA and CLIA are commercially available. Moreover, anti-SRP aAbs are well detected using IFI on Hep2 cells, a method that reveals the localization of autoantigens which are associated with specific staining patterns, making them easily detectable. SRP appears with a finely granular cytoplasmic staining making IFI on Hep2 cells an interesting element to support anti-SRP immunoassay results [26,27]. However, efforts towards harmonization and standardization among the different proposed methods are still necessary.

The assessment of the analytical and clinical quality of anti-SRP and anti-HMGCR tests was discussed by the ENMC and the European Autoimmunity Standardization Initiative (EASI) working groups [25,28]. It was noted that the comparison between anti-SRP-54 ELISA and IP revealed strong agreement between the methods with ELISA exhibiting high specificity (100%) and sensitivity (88%) while the comparison between line blot and ALBIA revealed 87% agreement. In contrast, immunoblot presented some false negative. Anti-HMGCR ELISA test showed a sensitivity of 94.4% and a specificity of 99.3% compared to IP. Furthermore, the evaluation using ELISA, CLIA and ALBIA on positive and negative sera for anti-HMGCR antibodies demonstrated an excellent concordance agreement (close to 100%) among these different methods. Immunoblots offer a broader screening capability, yet they remain limited to qualitative analysis. Monospecific assays, on the other hand, enable precise determination of antibody titers that can be a critical parameter as studies have indicated a correlation between aAb levels and disease severity [14].

IIM aAbs are more than diagnosis markers, they are also prognosis markers, of cancer for instance. For anti-HMGCR IMNM results were conflicting, suggesting a mild association with cancer [11,29,30]. No association was found for anti-SRP. In contrast seronegative IMNM was found at increased risk of malignancy [11]. More than just diagnosis and prognosis markers of the disease, anti-SRP and anti-HMGCR aAbs play a pathogenic role in IMNM.

## 4. Pathogenic Autoantibodies as Key Players in Mechanisms of Disease

In systemic lupus erythematosus (SLE), anti-dsDNA antibody levels were shown to be correlated with disease activity and their decrease led to the remission of patients, suggesting their pathogenicity [31]. In a similar manner, the pathogenic role of anti-SRP and anti-HMGCR antibodies was suggested when anti-SRP and anti-HMGCR antibody levels in sera were correlated with CK levels in sera, and muscle weakness indicating the extent of muscle breakdown and hence the severity of the disease [14,32]. Additionally, several studies demonstrated the efficacy of plasmapheresis or immunosuppressive treatments as prednisone or rituximab to reduce the number of antibodies, resulting in a recovery of muscle strength in patients [14,33,34]. All these data provide compelling evidence suggesting a pathogenic role of these antibodies.

The correlation between aAb titer and CK levels, along with the effects of depleting treatments suggests the involvement of autoantibodies. Experimental in vitro evidence further supports this hypothesis. Rojana-udomsart et al. demonstrated that the culture of human myoblasts in the presence of serum from patients with anti-SRP IMNM, with a high titer, resulted in a decrease in cell survival [35]. Subsequently, another study showed that plasma from patients with anti-SRP positive or anti-HMGCR positive IMNM led to a decrease in myotube surface area in culture. Similar results were obtained after incubating the myotubes with purified total IgG, as well as in the presence of purified aAbs against SRP or HMGCR. Furthermore, the expression of TRIM63/MURF1 and MAFbx/ATROGIN-1 mRNA, which are markers of atrophy, were increased. This increase appears to be linked to an upregulation of pro-inflammatory molecules such as IL6, TNF, and ROS, factors known to induce muscle atrophy [36]. Moreover, plasma, purified total IgG, or purified anti-SRP or anti-HMGCR aAbs from patients with IMNM decreased the fusion of myoblasts into myotubes reflecting a negative effect of these aAbs on in vitro muscle regeneration. The secretion of IL-4 and IL-13, known to play a major role in myotube formation from myoblasts, is also decreased. Myotube formation was then restored after the addition of these cytokines to the medium [36]. Thus, anti-SRP or anti-HMGCR aAbs have a direct pathogenic role in vitro by (i) reducing cell survival, (ii) inducing atrophy of already mature fibers, and (iii) preventing optimal regeneration from myoblasts.

The interaction between these aAbs and their target was investigated further by Romish et al. These authors studied the impact of anti-SRP54 on the function of the SRP54 protein and of the SRP complex in vitro. They found a significant and targeted inhibitory impact of anti-SRP54 aAbs on the in vitro translocation of the secretory protein preprolactin. These aAbs disrupt the binding of signal sequence of protein to SRP54, likely through steric hindrance. Moreover, the aAbs impeded the SRP receptor-mediated release of ER signal sequences from the SRP54 subunit [16]. To our knowledge, only the impact of anti-SRP54 was studied and not the other anti-SRP or anti-7SL RNA aAbs.

Unlike anti-SRP54 aAbs, which inhibit the function of the protein as presented above, the inhibitory activity of anti-HMGCR aAbs on protein function has not been yet demonstrated to our knowledge. In 2003, a study demonstrated that disruption of the HMGCR gene in mice was lethal, with the mice not surviving beyond the E8.5 stage underscoring the importance of this protein in embryonic development [37]. Additionally, it has been shown that a specific homozygous invalidation of the HMGCR gene in skeletal muscle induced postnatal myopathy, characterized by high levels of CK and muscle necrosis [38]. Thus, it appears that the absence of HMGCR in muscle is the root cause of the myopathy. Therefore, it is plausible to hypothesize that aAbs could inhibit the function of the protein and induce myopathy.

As in vitro studies demonstrated the impact of aAbs on muscle cells and to clarify the effects of aAbs, we evaluated their pathogenic role by (i) an active immunization of the animal with recombinant autoantigens, and (ii) a passive transfer of purified antibodies from patients with IMNM. First, immunization of mice with recombinant SRP and HMGCR proteins led to the detection of anti-HMGCR and anti-SRP antibodies in the serum on day 7 and day 28, respectively, and a decrease in grip strength and in situ muscle strength [39]. In the same way, the passive transfer of plasma or purified IgG from patients with IMNM, but not IgG-depleted plasma to mice, resulted in a decrease in grip strength and muscle strength in mice. This muscle impairment was associated with an increased number of necrotic myofibers and macrophage infiltration [39]. This active and passive immunization demonstrated that anti-SRP and anti-HMGCR antibodies were pathogenic in vivo. This murine model could be useful in understanding further the pathophysiological mechanisms. However, it has several limitations. Indeed, the murine model requires the administration of purified patient antibodies for several consecutive days to develop the disease [39]. These purified antibodies come from patient plasmapheresis, which is a limitation as it is a finite resource. The development of recombinant antibodies produced in cells could be an interesting prospect to obtain a robust and sustainable murine model. Furthermore, another limitation of this model is that the mice receive an immunosuppressant injection to prevent xenogeneic responses towards the injected human-origin IgG. However, this injection eliminates immune cells that could potentially play a role in the pathological reaction. It would be interesting to consider using animals that are tolerant to human IgG.

Knowing that aAbs have a pathogenic effect, we sought to understand their mechanism of action. The hypothesis that the complement system could play a role in the physiopathology of IMNM has been highlighted. Initially, it was observed in muscle biopsies from patients’ complement deposits at the sarcolemma of non-necrotic muscle fibers [40]. Moreover, aAbs associated with IMNM are predominantly of the IgG1 isotype [14], an isotype known to activate the complement system. These observations suggest a mechanism mediated by complement-dependent antibodies. To determine the involvement of complement in myolysis, human myoblasts were cultured in the presence of plasma from patients with SRP-positive IMNM, and a decrease in cell survival was observed [35]. Interestingly, this survival was even more affected when human complement was added to the culture [35]. Furthermore, a co-localization of SRP and C3c at the cell membrane on myoblasts was observed after pre-incubation with serum from patients with anti-SRP positive IMNM. Additionally, a positive staining for membrane attack complex C5b-9 was observed [35]. Finally, in vivo, it has been demonstrated that the administration of total IgG from patients with anti-SRP positive or anti-HMGCR positive IMNM to complement-deficient mice does not induce muscle deficit. Moreover, supplementation with human complement in mice worsens the muscle deficit in the presence of total IgG from patients. Associated with this muscle deficit, necrotic fibers and infiltration of macrophages were observed in muscle biopsies from mice and complement deposits were detected within the sarcolemma of diseased mice [39]. All these findings suggest the involvement of the complement system in antibody-mediated pathogenicity in patients and bring new keys to develop more specific therapeutic strategies for patients.

SRP and HMGCR are intracellular and ubiquitous targets. To date, it is still unknown how these antibodies manage to specifically target these proteins, and why this leads to muscle-related manifestations exclusively. A first hypothesis includes the possibility that antibodies may enter cells to reach their target. It has indeed been described that the TMab4 antibody, which is a human IgG1, can access the cytosol of cells. This penetration is possible after interaction with the heparan sulfate proteoglycan (HSPG) located on the surface, acting as a receptor. This complex is then internalized into endosomes. The acidification of the endosome induces conformational changes in the antibody due to a specific sequence located on the CDR3 light chain. These conformational changes lead to an interaction with the endosomal membranes, resulting in the formation of a pore allowing the antibody to escape [41]. Although the phenomenon of intracellular penetration is currently poorly documented, it should not be ruled out in the case of aAbs in IMNM. Another hypothesis proposed by Allenbach et al. is a potential ectopic expression of these proteins on the cell membrane, which would make them accessible to antibodies following labeling on muscle cells. However, this labeling has not been observed under a confocal microscope with co-labeling using a strictly membrane-bound protein [40]. On the other hand, complement is partly involved in IMNM pathophysiology while it is not expected to act on antibodies bound to intracellular targets, suggesting an extracellular location of the autoantigens. Finally, it is also possible to suspect that anti-SRP or anti-HMGCR antibodies may recognize another protein, based on sequence homology, located on the membrane. Further research with a deeper molecular or cellular exploration of their pathogenic mechanisms is needed to enhance the understanding of IMNM.

## 5. B Cell and IgG Targeted Therapies as Therapeutic Perspectives

The first and crucial aspect in managing IMNM lies in their diagnosis. However, despite numerous international workshops aimed at defining the inclusion criteria for this disease, there is a lack of global harmonization. Indeed, in 2023, it is still possible to find clinical trials that rely on the Bohan and Peter classification from 1975 that does not consider IMNM but rather PM. This heterogeneity in the definition of IMNM poses a barrier to their diagnosis and, consequently, to their therapeutic management, particularly concerning clinical trials.

To slow down the course of the disease, some therapies can be implemented. The goal of those therapies is to reduce muscle inflammation and necrosis and promote myofiber regeneration. Some indicators can be monitored to assess treatment efficiency. First, clinical score of muscle strength such as MMT-8 (Manual Muscle Testing) [42]. Additionally, muscle MRI follow-up can be useful to monitor disease activity with a focus on edema, muscle atrophy or fat replacement [43]. Another monitoring tool is muscle enzyme blood levels such as CK, aldolase and lactate dehydrogenase (LDH). These markers are indicators of muscle cell lysis. In IMNM treatment, the aim is to reduce muscle inflammation characterized by normal levels of muscle enzymes and normal muscle strength or at least functional status.

An absence of recommendations for the treatment of IMNM complicates the conduct of clinical trials. However, some elements may emerge through the study of long-term cohorts. In the absence of official guidelines, treatment relies on expert opinions. The primary treatment approach involves administering corticosteroids at a dose of 1 mg/kg/day, along with an immunosuppressant, usually methotrexate at a dose of 0.3 mg/kg of body weight per week, for one month. Corticosteroids are favored as the initial treatment due to their rapid action and broad impact on inflammatory diseases. However, corticosteroids have significant side effects, leading some experts to consider corticosteroid-sparing regimens using alternative immunosuppressants [44]. If successful, corticosteroids are typically gradually tapered in favor of methotrexate.

For more severe cases, corticosteroid/methotrexate combination therapy may be supplemented with IVIg (2 g/kg/month) or plasmapheresis. Plasmapheresis, aimed at removing pathogenic aAbs from the plasma, is now less used and even less recognized, especially in the U.S. It serves as a short-term solution primarily considered for bedridden patients to provide temporary relief, showing promising results, particularly in patients with anti-SRP positive and HMGCR positive IMNM [45]. Patients with anti-SRP positive IMNM may also receive rituximab, sometimes as a first line, although its efficacy appears lower or non-existent in patients with anti-HMGCR positive IMNM, even though it has demonstrated interesting outcomes in about one-third of patients with refractory anti-HMGCR IMNM [46].

In case of relapse, other immunosuppressants such as azathioprine, mycophenolate mofetil, cyclophosphamide, or cyclosporine may be considered [47].

For a broader view on which treatments have been tested or are being tested in formal clinical trials, a review on clinicaltrials.gov was performed. Despite the inclusion criteria for this disease, the lack of global harmonization has led to the establishment of several clinical trials based on Bohan and Peter’s 1975 classification, which considers PM rather than IMNM. Consequently, we choose to exclude PM in our research. We used the following key words: condition/disease “immune mediated necrotizing myopathy” and “idiopathic inflammatory myopathy” by checking that the inclusion criteria for IIM included anti-SRP or anti-HMGCR antibodies. 17 treatment interventional clinical trials were identified (Table 1). Among these 17 trials, 1 was excluded because they evaluated non-pharmacological interventions which was not the focus of this review. We detail below the different treatment targets used, classifying them into four different groups: treatments targeting aAbs, treatments targeting immune cells (B cells and others), treatments targeting pro-inflammatory cytokines and other treatments.

### 5.1. Treatments Targeting aAbs

In this first category we chose to include Intravenous Immunoglobulins (IVIg), even though the mechanism of action may be broader. Two main mechanisms have been suggested and can coexist: first, an immunomodulatory effect and then, a saturation of neonatal Fc receptor (FcRn), a receptor known to have an affinity for the Fc fragment of IgG and responsible for their recycling [48]. This line of treatment was evaluated in several clinical trials using different brands of IVIg such as GB-0998^®^ or Nanogam^®^ at standard courses of 2 g/kg over 2 to 5 days. Completed clinical trials using previous definitions of IIM concluded on the superiority of IVIg compared to placebo [49] but a more recent trial with a more recent definition as inclusion criteria is still ongoing [50].

In the same category, we will find the anti-FcRn drug class. Efgartigimod is a human IgG1 Fc fragment mutated to improve its affinity for FcRn. Nipocalimab, also known as JNJ-80202135, is a fully human deglycosylated IgG1λ monoclonal antibody engineered to have no Fc effector potential [51]. Both bind to FcRn inhibiting IgG recycling and lowering systemic IgG, including pathogenic IgG aAbs. By its ability to reduce the levels of pathogenic IgG, Efgartigimod has shown clinical efficacy in aAb-mediated diseases. For example, it led to the improvement of myasthenia gravis (MG) symptoms in a phase 3 study [52]. Preclinical studies in an IMNM mouse model showed the efficacy of efgartigimod, justifying a therapeutic trial in patients [53]. Both treatments, Efgartigimod and Nipocalimab, are currently in phase 2/3 randomized, placebo-controlled clinical trials. Hence their efficacy in IMNM are not yet known. Besides Efgartigimod or Nipocalimab, other FcRn inhibitors are available. Rozanolixizumab is a humanized IgG4 monoclonal anti-human FcRn antibody that efficiently reduced IgG in FcRn transgenic mouse model and cynomolgus monkey [54]. These treatments may lead to side effects such as hypogammaglobulinemia and hypoalbuminism. However, recent studies have demonstrated the safety and tolerability of FcRn inhibitors [55]. Indeed, in phase 3 trial NCT03669588 in MG patients, efgartigimod administration resulted in a higher risk of airway and urinary tract infections compared to placebo, although these effects were described as mild to moderate in severity [52]. Besides, serum albumin (SA) was not decreased in a phase 1 trial in healthy volunteers [56]. In a phase 2 study of efgartigimod in ITP, SA concentration was not decreased compared to placebo [57]. Similar results were observed in a phase 2 trial in pemphigus foliceus and in a phase 3 trial in MG [52,58]. A caveat for the use of FcRn inhibitors may be the non-selective reduction of all IgG levels, including protective antibodies with immunosuppressive effects. Newly developed engineered recombinant antigen-Fc fusion proteins called seldegs (for selective degradation) have been shown to induce lysosomal trafficking of antigen-specific aAbs and their specific elimination, minimizing off target effects [59]. This approach led to disease amelioration in murine experimental autoimmune encephalomyelitis exacerbated by the administration of patient-derived myelin oligodendrocyte glycoprotein-specific antibodies, without affecting antibodies with other antigen specificities [60]. However, the use of this methodology requires knowledge of the specific aAbs to be targeted and this approach could not be used for the 10 to 12% of seronegative patients. In-depth studies should therefore be conducted to identify new aAbs in the serum of these patients and to develop new therapeutic drugs.

Lastly, we include anti-complement therapy in this category, as it is dependent on aAbs. Eculizumab, a C5 inhibitor is largely used in ab-mediated disease like severe resistant lupus nephritis [61] or neuromyelitis optica [62] and has already successfully treated patients, At the time of writing this review, one molecule has been tested in IMNM: zilucoplan. It is a macrocyclic peptide that binds to the human C5 fraction of complement and inhibits its cleavage into C5a and C5b thus inhibiting the formation of the membrane attack complex. Although complement activation has been shown to play a role in IMNM in vitro [35] and in vivo [39], zilucoplan showed no improvement in CK levels in patients with IMNM compared to the placebo group in a multicenter, randomized, double-blind phase 2 clinical trial [63]. However, it is plausible to suspect that some of the patients in the study were already resistant to previous treatments and had reached an advanced stage of the disease. In a murine model of IMNM, zilucoplan was shown to be effective in preventing the disease but not in a curative setting [64]. This suggests that this therapy could potentially offer more significant benefits in the early stages of the disease. Despite this negative clinical result, targeting complement factors at other levels in the cascade remain options to be tested.

### 5.2. Treatments Targeting Immune Cells

In this second category, we chose to include drugs targeting cells producing aAbs or playing a role in their production.

The first drug in this category are drugs targeting B cells. Rituximab, a chimeric monoclonal anti-CD20 antibody, is the first drug in this class to have proven its efficacy in refractory PM [65,66]. Rituximab has also been compared to cyclophosphamide in a phase 2b trial for patients with connective tissue disease-associated interstitial lung disease in the UK in which it showed a similar efficacy but fewer adverse events [67]. Based on these results, other classes of drugs have started clinical development such as belimumab, a fully human anti-BAFF (B-cell activation factor) monoclonal antibody. Belimumab has shown no new safety concerns in patients with refractory IIM compared to known adverse events in currently authorized indications such as SLE. However belimumab in monotherapy did not show significant efficacy compared to placebo in long term follow-up (40 weeks) of refractory IIM [68].

In clinical practice, it is observed that rituximab works well in patients who test positive for anti-SRP IMNM but less so in those with anti-HMGCR. The reasons for this aren’t fully known. However, it is possible that tissue B lymphocytes or plasma cells evade rituximab treatment. Recent studies have demonstrated the efficacy of anti-CD19 CAR T cell therapies in the treatment of SLE in a murine model and then in patients [69,70]. Moreover, in a study with a case of refractory ASA [71], it suggests that CAR T cells have a therapeutic effect after rituximab failure in this form of IIM. In view of these results, and given their mechanism of action, CAR T cell therapies could be a promising indication in the treatment of refractory IMNM. Indeed, several advanced therapy medicinal products (ATMP) have started clinical development in IMNM with anti-CD19 CAR-T (pCAR-19B and CABA-201) and anti-BCMA CAR-T cells (CT103A cells) but also universal allogenic CAR-T (BRL-301) with a target not yet detailed. All three ATMP are currently in phase 1 trials. CAR-T therapy safety data have been mainly constituted in the context of cancer patients. Acute adverse events such as cytokine release syndrome (CRS) and immune effector-cell associated neurotoxic syndrome (ICANS) are well described and management guidelines have been developed. Recently, FDA has reported a safety warning regarding the risk of T-Cell malignancy in long term follow-up of all currently approved product of this class (anti-BCMA and anti-CD19) [72]. The benefit/risk balance in the context of auto-immune diseases is different from cancer and a careful selection of eligible patients will be necessary given the need for a lymphodepleting conditioning before CAR-T cell administration, the risks of CRS and ICANS, and the potential consequence of B cell aplasia if prolonged. Preliminary safety data of those new ATMP remain scarce in auto-immune disease patients. For example, CT103A in refractory AQP4-IgG neuromyelitis optica have been tested on 12 patients and reports a safety profile similar to other approved CAR-T with a drug-free remission for eleven patients in a short follow-up. A long-term remission still needs to be confirmed as well as a controlled comparison [73]. Similarly, daratumumab (an anti-CD38 targeting plasma cells in multiple myeloma) could represent a therapeutic avenue if rituximab fails to deplete the entire B-cell compartment in patients with IMNM. However, to our knowledge, it has not yet been tested for this purpose.

Other clinical developments have focused on antigen-presenting cell (APC) activation. Abatacept, a soluble CTLA-4 analog that prevents APC from delivering the co-stimulatory signal, has been tested in phase 2 and 3 settings. Phase 2 trials showed clinical efficacy of abatacept [74] and ancillary study indicates that CD4/CD8 ratio may be a predictor of treatment efficacy [75]. However, the phase 3 trial failed to meet study objectives.

Finally, regulatory T cells (Treg) represent another target to induce tolerance. Low dose IL-2 supplementation has been shown to achieve clinical efficacy in rheumatoid arthritis, lupus erythematosus and other auto-immune diseases [76]. These data have prompted the development of trials for patients with IMNM. In this study patients supplemented with low dose IL-2 improved compared to baseline. However, the study lacked a control group to confirm efficacy [77].

Even if it has not been already tested in diagnosed IMNM patient in clinical trial, other drugs in this category can be broad spectrum immunosuppressors that target large panels of immune cells. We can cite mycophenolate mofetil (MMF), tacrolimus, cyclophosphamide and methotrexate as examples. Their broad action on the immune system comes with a higher risk of adverse events. Tacrolimus and methotrexate have been assessed in clinical settings. Methotrexate did not show a superiority in either safety or efficacy compared to glucocorticoids in early disease of PM and DM [78]. Tacrolimus was tested in a single-arm trial in PM associated with ILD and even though the study showed an encouraging safety profile, the study lacked a controlled arm to confirm safety and efficacy [79]. Finally, Zetomipzomib (KZR-616), a first-in-class immunoproteasome inhibitor with an immunomodulatory action on B cells, T cells and macrophages is being tested in a completed phase II randomized controlled trial, but the results are not yet published. Known data on healthy volunteers confirmed results obtained in preclinical studies but safety data were not discussed [80].

Plasmacytoid dendritic cells (pDC) can represent another cellular target. They have been shown to play a role in autoimmune diseases such as lupus erythematosus [81]. Daxdilimab, also known as MEDI 7734, is an anti-ILT7, a cell surface molecule specific to pDC. Daxdilimab underwent phase 1 trials in patients with DM and PM and presented an acceptable safety profile. However, subsequent phase 2/3 trials did not include patients with a clear IMNM diagnosis [82].

### 5.3. Treatments Targeting Inflammatory Cytokines

Several monoclonal antibodies targeting different types of pro-inflammatory cytokines have been or are being tested in myositis. We can cite drugs targeting IL-1 receptor, STAT3, TNFα, IL-6, TLR7, IL-12/23 and interferon β. Among completed trials several anti-inflammatory cytokines did not show efficacy in PM such as anti-IL-1 receptor (anakinra), anti-TNFα (infliximab), anti-IL-6R (tocilizumab) and anti-IL-12/23 (ustekinumab).

One antibody that showed efficacy in a placebo-controlled trial including patients with PM or DM was sifalimumab, an anti-interferon α fully human monoclonal antibody [83]. On the one hand this was a phase 1b trial with a small cohort of patients and these data need to be completed with a larger cohort. On the other hand, sifalimumab development has been stopped in favor of another anti-interferon α, anifrolumab, but no trials including patients with PM or IMNM are in process. It has recently been demonstrated that TLR7 was upregulated at the transcript level in muscle tissue of patients with IMNM [84], which could participate in the pathogenesis. TLR7 could therefore be an interesting target for new therapeutics. Moreover a case report indicated efficacy of anifrolumab in one IVIg refractory DM patient [85], while a clinical trial with anifrolumab in DM is still ongoing (NCT05669014).

### 5.4. Other Treatments

This last subgroup comprises drugs with heterogeneous mechanisms of action through non immunological pathways but may complete a therapeutic arsenal to restore muscle tissue.

The first one, is nintedanib, a tyrosine kinase inhibitor of several factors like fibroblast growth factor (FGF) and vascular endothelial growth factor (VEGF). Nintedanib possesses EMA and FDA approval in interstitial fibrotic pneumopathy and is currently in several observational trials in myositis associated ILD. The antifibrotic properties of nintedanib have primarily been investigated in lungs. However, in vivo investigations have shown that nintedanib antifibrotic properties also affect muscle tissue which could be relevant for muscle regeneration in patients with IMNM [86].

Another drug that could be interesting is a candidate in development that is a Glucagon Like Peptide-1 (GLP-1) agonist. GLP-1 regulates muscle remodeling [87]. Moreover, the froniglutide, a GLP-1 agonist has improved inflammatory myopathies in in vitro and in vivo models of PM [88].

Finally, a last treatment pathway is altered mitochondrial function. Mitochondria play a role in pro-inflammatory signals and other pro-inflammatory markers may exert mitochondrial oxidative stress. Thus, a strategy to reduce mitochondrial oxidative stress may represent a therapeutic treatment as well as a preventive action [89]. Based on these elements, a team developed human umbilical cord mesenchymal stem cell-derived mitochondria (PN-101), tested them in vitro and in vivo and succeeded at reducing inflammatory response [90]. A phase 1 and 2 study including patients with refractory PM and DM has yet to demonstrate safety and efficacy in clinical settings.

## 6. Conclusions

To conclude, the discovery and the identification of autoantibodies has revolutionized the classification of IIM and, consequently, the diagnosis of patients with IMNM. Although current diagnostic techniques are a crucial component of patient management, there is still room for improvement. Recent advances in demonstrating the pathogenicity of antibodies through in vitro and in vivo experiments have provided a rational basis for exploring new therapeutic approaches. However, extensive research is still required to understand how these antibodies reach their target and whether they could inhibit the function of SRP and HMGCR only in muscle. Finally, research and discovery of a possible new target in patients with seronegative IMNM would also open up the field for new therapeutic approaches.

## Figures and Tables

**Table 1:** Clinical trials obtain on clinicaltrials.gov with key words “IMNM” and “IIM” with anti-SRP and/or anti-HMGCR as inclusion criteria in December 2023. NA: not applicable.

N° Trial	Sponsor	Mono/ Multicentric	Phase	Molecule	Number of Patients	Status	Start Date	Completion Date
NCT05832034	Academisch Medisch Centrum—Universiteit van Amsterdam	Monocentric	Phase 2	IVIg	48	Recruiting	13 September 2021	-
NCT04450654	University of Washington	Monocentric	Phase 2	IVIg		Withdrawn	1 May 2022	25 July 2022
NCT05523167	ArgenX	Multicentric	Phase 2/3	Efgartigimod	240	Recruiting	12 October 2022	-
NCT05979441	ArgenX	Multicentric	Phase 3	Efgartigimod	240	Recruiting	12 September 2023	-
NCT05379634	Janssen Research & Development, LLC	Multicentric	Phase 2	Nipocalimab	200	Recruiting	5 July 2022	-
NCT04025632	Ra Pharmaceuticals	Multicentric	Phase 2	Zilucoplan	27	Completed	7 November 2019	14 June 2021
NCT00774462	Assistance Publique—Hôpitaux de Paris	Monocentric	Phase 2	Rituximab	30	Completed	1 January 2008	1 December 2011
NCT02347891	Northwell Health (New York)	Monocentric	Phase 2/3	belimumab	60	Unknown status	1 January 2015	-
NCT06056921	Chongqing Precision Biotech Co., Ltd.	Monocentric	Phase 1	CAR-T CD19	24	Recruiting	31 August 2023	-
NCT04561557	Tongji Hospital	Monocentric	Phase 1	CT103 Cells (CAR-T anti-BCMA)	18	Recruiting	22 September 2020	-
NCT05859997	Bioray Laboratories	Monocentric	Phase 1/2	UCAR-T BRL-301 (anti-BCMA)	15	Recruiting	17 May 2023	-
NCT06154252	Cabaletta Bio	Monocentric	Phase 1/2	CAR-T CD19 (CABA-201)	18	Recruiting	17 November 2023	-
NCT02971683	Bristol-Myers Squibb	Multicentric	Phase 3	Abatacept	149	Terminated	4 April 2017	2 August 2022
NCT05799755	University of Pittsburgh	Multicentric	Phase 4	Nintedanib	134	Recruiting	1 August 2023	-
NCT03092180	University Sao paulo	Monocentric	Observationnal	Glucocorticoid	60	Recruiting	1 January 2005	-
NCT04062019	Peking University People’s Hospital	Monocentric	Phase 2	IL-2	15	Recruiting	30 August 2019	-
NCT04486261	Rigshospitalet, Denmark	Monocentric	NA	Non pharmacological	34	Active not recruiting	30 August 2021	-

## Data Availability

Not applicable.
